# Syntheses and a Solid State Structure of a Dinuclear Molybdenum(V) Complex with Pyridine 

**DOI:** 10.3390/ma3010150

**Published:** 2010-01-04

**Authors:** Barbara Modec, Jon Zubieta

**Affiliations:** 1 Department of Chemistry and Chemical Technology, University of Ljubljana, Aškerčeva 5, 1000 Ljubljana, Slovenia; 2 Department of Chemistry, Syracuse University, Syracuse, New York, NY 13244, USA

**Keywords:** polyoxomolybdates, metal−metal bonded {Mo_2_O_4_}^2+^ core, molybdenum(V)

## Abstract

A mononuclear complex [MoOCl_4_(H_2_O)]^−^ readily forms a metal−metal bonded {Mo_2_O_4_}^2+^ core. A high content of pyridine in the reaction mixture prevents further aggregation of dinuclear cores into larger clusters and a neutral, dinuclear complex with the [Mo_2_O_4_Cl_2_(Py)_4_] composition is isolated as a product. Solid state structures of two compounds containing this complex, [Mo_2_O_4_Cl_2_(Py)_4_]·2.25Py (**1**) and [Mo_2_O_4_Cl_2_(Py)_4_]·1.5PyHCl (**2**), were investigated by X-ray crystallography.

## 1. Introduction 

**Scheme 1:**
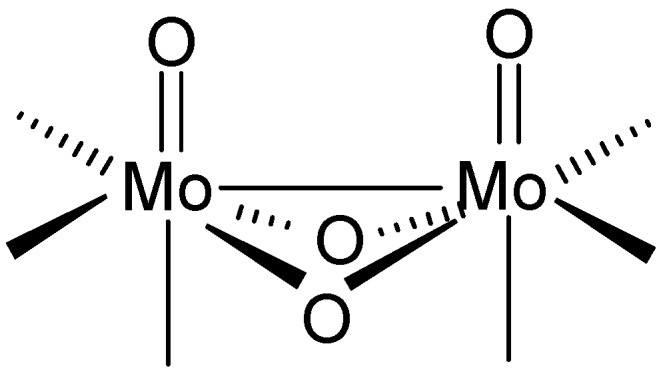
A dinuclear {Mo_2_O_4_}^2+^ structural core.

The {Mo_2_O_4_}^2+^ core (shown in Scheme 1) with *d* electrons localized in a Mo(V)−Mo(V) single bond appears as a recurrent structural motif in many contexts of molybdenum(V) coordination chemistry [1,2,3,4,5,6,7]. A group of high-nuclearity oxomolybdenum(V) complexes whose structures may be seen as the assemblies of two or more {Mo_2_O_4_}^2+^ units has emerged in the past two decades [8]. The aggregation of dinuclear units is made possible by the ability of oxide and alkoxide ions to participate in μ_2_-, μ_3_- or even μ_4_-bridging interactions. The oxomolybdenum(V) clusters are of interest because of their intermediacy between the oligomeric alkoxides and the polymeric oxides on the other side. The expectation is that their chemistry may provide some insight into the catalytic processes taking place on the surface of the solid metal oxides [9].

(PyH)_5_[MoOCl_4_(H_2_O)]_3_Cl_2_ with a mononuclear [MoOCl_4_(H_2_O)]^−^ ion has been shown to serve as a suitable starting material in the preparation of {Mo_2_O_4_}^2+^-containing clusters [10]. Changes in the contents of reaction mixtures or reaction conditions resulted in clusters with different nuclearities or different connectivities among the dinuclear building blocks. Two, three, four or up to six dinuclear units were linked through bridging oxides and/or methoxides to form tetranuclear, hexanuclear, octanuclear, decanuclear and dodecanuclear clusters with the [Mo_4_O_8_(OMe)_2_Cl_2_(Py)_4_], [Mo_6_O_12_(OMe)_6_(Py)_4_], [Mo_8_O_16_(OMe)_8_(Py)_4_], [Mo_10_O_26_(Py)_8_] and [Mo_12_O_28_(OMe)_2_Cl_2_(3-MePy)_8_] compositions [11,12,13]. The subtle nature of the aggregation is illustrated by the assembly of four {Mo_2_O_4_}^2+^ units which was seen to produce three different architectures: (i) a cyclic {Mo_8_O_8_(μ_2_-OMe)_8_(μ_2_-O)_8_} core, and two cores with more condensed structures, (ii) {Mo_8_O_8_(μ_3_-OMe)_2_(μ_2_-OMe)_4_(μ_3_-O)_4_(μ_2_-O)_4_}^2+^ [12] and (iii) {Mo_8_O_8_(μ_3_-O)_4_(μ_2_-O)_8_} [14]. The presence of methanol in the reaction mixture was found to be crucial for the aggregation process. The possible role that methanol or alcohols in general play in the formation of dinuclear units and their further aggregation will be discussed presently. In the absence of alcohol, (PyH)_5_[MoOCl_4_(H_2_O)]_3_Cl_2_ produced in the mixture of pyridine and a non-coordinating solvent two compounds which both contained a dinuclear {Mo_2_O_4_}^2+^-based complex with the [Mo_2_O_4_Cl_2_(Py)_4_] composition. Herein, the syntheses and the solid state structures of [Mo_2_O_4_Cl_2_(Py)_4_]·2.25Py (**1**) and [Mo_2_O_4_Cl_2_(Py)_4_]·1.5PyHCl (**2**) are presented. 

## 2. Results and Discussion

The metal−metal bonded, dinuclear {Mo_2_O_4_}^2+^ core is a result of the facile substitution chemistry on the mononuclear [MoOCl_4_(H_2_O)]^−^ ion. In spite of the many known compounds possessing the dinuclear structural core, the details of its formation remain unknown. Undoubtedly, one of the key prerequisites is the presence of water. Results of our previous work show that methanol actively participates in substitution and/or dimerization reactions through the coordination of the methoxide ions formed *in-situ*. The coordinated methoxide can in turn react with water to form an oxido ligand. A direct evidence of the latter process is the observed transformation of [Mo_2_O_3_(OMe)(OOCCH_3_)Cl_4_]^2−^, a complex with a bridging methoxide, to the {Mo_2_O_4_}^2+^-containing products [15]. The formation of {Mo_2_O_4_}^2+^ species was found to be faster and more reproducible when methanol was present in the reaction mixture. Furthermore, it has also been observed that the reaction mixtures containing large amounts of methanol or other alcohols tend to produce higher-nuclearity clusters with either oxido or alkoxido ligands shared between the constituent dinuclear units. In such clusters, the relative content of the chlorido ligands was found to be rather small. Conversely, the reaction systems lacking methanol do not favour further association of dinuclear cores and as such provide a convenient environment for the isolation of the {Mo_2_O_4_}^2+^ prototypes. The reaction system consisting solely of molybdenum(V) starting material and pyridine exemplifies such a medium. When pyridine occupies four out of six available coordination sites of the {Mo_2_O_4_}^2+^ core, a neutral [Mo_2_O_4_Cl_2_(Py)_4_] complex is formed. It is of interest to note that [Mo_2_O_4_Cl_2_(Py)_4_] crystallizes only with the incorporation of either a huge amount of pyridine solvent molecules (compound **1**) or pyridinium chloride(compound **2**). None of the two compounds is stable when removed from the mother liquor. Compound **1** dissolves almost instantaneously in its own solvent molecules. 

**Figure 1:**
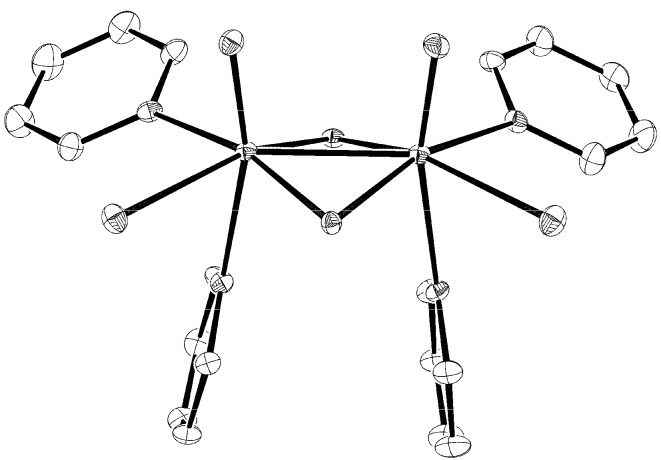
An ORTEP drawing of [Mo_2_O_4_Cl_2_(Py)_4_] molecule in **2**. Atoms are represented by displacement ellipsoids at the 30% probability level. Hydrogen atoms were omitted for the purpose of clarity.

As shown by the X-ray structure analysis, **1** and **2** are both [Mo_2_O_4_Cl_2_(Py)_4_]-containing compounds (Figure 1) which differ in the content of the solvent molecules of crystallization or counter ions. For both, the asymmetric unit contains two crystallographically independent complex molecules with very similar structural parameters (Table 1). 

**Table 1:** Selected bond lengths (Å) and angles (°) in [Mo_2_O_4_Cl_2_(Py)_4_] molecules of **1** and **2**.^[a] ^

	1		2	
**Mo−Mo**	2.5502(8)	2.5517(8)	2.5427(6)	2.5441(6)
**fold angle^[b]^**	144.5(2)	144.5(2)	143.0(1)	143.4(1)
**Mo−Cl**	2.465(2), 2.467(2)	2.452(2), 2.477(2)	2.457(1), 2.460(1)	2.453(1), 2.466(1)
**Mo−N(Py)**	2.272(5) *vs*.	2.276(5) *vs*.	2.261(4) *vs*.	2.256(5) *vs*.
	2.397(5)	2.384(5)	2.388(9)	2.382(4)
	2.265(5) *vs*.	2.261(5) *vs*.	2.263(4) *vs*.	2.276(4) *vs*.
	2.407(5)	2.396(5)	2.381(4)	2.381(4)

^[a]^ Two sets of parameters, one for each molecule in the asymmetric unit. ^[b]^ Defined as a dihedral angle between two Mo(μ_2_-O)_2_ planes. The smaller the fold angle, the greater the deviation of the Mo(μ_2_-O)_2_Mo bridging unit from the planarity.

The overall features of the basic {Mo_2_O_4_}^2+^ core are as determined previously in related compounds [8]: (i) a short distance between molybdenum atoms, 2.5502(8) and 2.5517(8) Å for **1** and 2.5427(6) and 2.5441(6) Å for **2**, typical for a single metal−metal bond; (ii) a puckered Mo(μ_2_-O)_2_Mo ring with the metal atoms above and the bridging oxygen atoms below the mean plane. The rather short metal−metal bond lengths are due to the high content of pyridine ligands whose nature is electron-donating. The dihedral angle between the two Mo(μ_2_-O)_2_ planes is by *ca*. 10° smaller than the angles observed in a series of oxalato complexes of the [Mo_2_O_4_(η^2^-C_2_O_4_)_2_(R-Py)_2_]^2−^ (R-Py = alkyl-substituted pyridine) type [16]. A distorted octahedral coordination of each metal centre of the {Mo_2_O_4_}^2+^ core is completed by a chloride at 2.452(2)−2.477(2) Å for **1** and at 2.453(1)−2.466(1) Å for **2** and a pair of pyridine ligands with two distinctly different bond lengths. A short one occupies the 2.256(5)−2.276(5) Å range, whereas a long one the 2.381(4)−2.407(5) Å range. The reason lies in one of the two pyridine ligands being bound to a position *trans* relative to the Mo=O group and being, therefore, subject to its well-documented *trans* influence [17]. The lengthening of the bonds *trans* to the terminal oxide is a general phenomenon found throughout the {MoO}^3+^ compounds. The molybdenum-to-pyridine bond lengths in **1 **and **2** are shorter than the ones found in [Mo_2_O_2_(μ_2_-S)_2_{S_2_P(OEt)_2_}_2_]∙2Py [18]. The latter compound exemplifies an extreme case of *trans* influence of the molybdenyl group: because of the extreme bond length, 2.545(7) and 2.569(7) Å, the compound is considered as an adduct with pyridine. The extent to which the *trans* influence is expressed within a certain complex differs from one case to another. [{MoOCl_2_(Py)_2_}_2_O], another dinuclear molybdenum(V) complex, with a pair of pyridine ligands being *trans* to each other displays Mo−N bonds with lengths of 2.169(5)−2.209(5) Å [19]. Their length may be explained in terms of a smaller *trans* influence of pyridine as compared to that of a bridging oxide in **1** and **2**. The non-coordinated chlorides in **2** participate in hydrogen-bonding interactions with pyridinium cations. The Cl···N distances are in the 2.951(4)−3.200(7) Å range [20].

**Figure 2:**
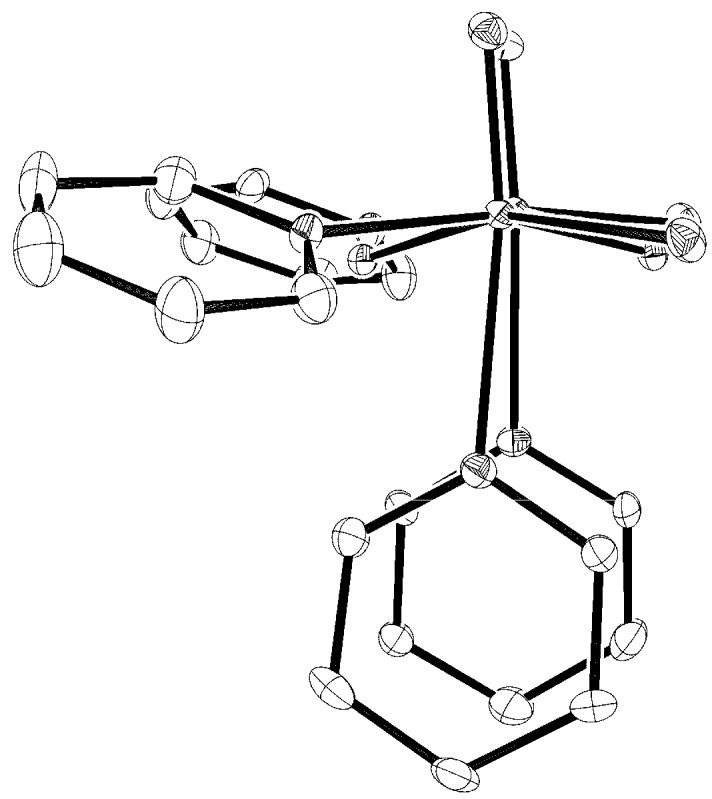
A view along the metal−metal bond vector.

## 3. Experimental Section 

### 3.1. General

All procedures were conducted in air. Pyridine, methanol, acetonitrile and diethyl ether were purchased from Aldrich and used without further purification. (PyH)_5_[MoOCl_4_(H_2_O)]_3_Cl_2_ was prepared following the published procedure [21]. IR spectra were measured on solid samples as Nujol mulls using a Perkin Elmer 2000 series FT-IR spectrometer.

### 3.2. Preparation of [Mo_2_O_4_Cl_2_(Py)_4_]·2.25Py *(**1**)*

(PyH)_5_[MoOCl_4_(H_2_O)]_3_Cl_2_ (2.5 g, 5.83 mmol of Mo) was added to pyridine (20 mL). One third of the reaction mixture was transferred to a 10 mL glass tube. The tube was sealed and heated at 130 °C for 100 h under autogenous pressure. After heating, the reaction vessel was allowed to cool slowly to room temperature. Large, orange block-like crystals of **1** formed after a period of four days. 

### 3.3. Preparation of [Mo_2_O_4_Cl_2_(Py)_4_]·1.5PyHCl *(**2**)*

(PyH)_5_[MoOCl_4_(H_2_O)]_3_Cl_2_ (0.125 g, 0.29 mmol of Mo) was added to the mixture of acetonitrile (5 mL) and pyridine (5 mL). After 10 minutes, the initially dark brown solution changed its colour to deep orange. Diethyl ether (approximately 20 mL) was added dropwise, until the first cloudiness was observed. Upon mixing, the solution became clear again. A clear solution was left to stand in a closed Erlenmeyer flask at ambient conditions. Clusters of orange, block-like crystals appeared within 24 hours. Yield: 70 mg (59%). IR (cm^–1^): 1,632vvs, 1,605vvs, 1,570m, 1,532vvs, 1,450vvs, 1,348m, 1,322m, 1,279w, 1,252s, 1,216vs, 1,149vs, 1,065vs, 1,042vvs, 1,012vvs, 1,001vvs, 942vvs, 722vvs, 641vs, 632vs, 606vs, 485s, 435s. 

### 3.4. X-ray crystallography

Each crystal was mounted on the tip of a glass fibre with a small amount of silicon grease and transferred to a goniometer head. Data were collected on a Bruker SMART CCD (compound **1**) or Nonius Kappa CCD (compound **2**) diffractometer. SHELXL-97 was employed for the structure solution and refinement [22]. The high content of pyridine solvent molecules in **1** resulted in large *R*1 and *wR*2 residuals. One of the pyridine solvent molecules was located with its centre of gravity on the inversion centre. Although the disorder was resolved using a PART −1 instruction, the displacement parameters of its atoms remained large. The structure of **2** was solved and successfully refined in a non-centrosymmetric *P* 2_1_ space group. When refined in a centrosymmetric *P* 2_1_/*c* space group, a small fragment of the structure, a pyridinium cation with a hydrogen-bonded chloride counter anion, was located on the inversion centre. Its disorder could not be resolved. Figures depicting the structures were prepared by Ortep-3 [23] and CrystalMaker [24]. Cell parameters and refinement results are summarized in Table 2. Further details on the crystal structure investigations may be obtained from The Cambridge Crystallographic Data Centre via www.ccdc.cam.ac.uk/data_request/cif by quoting the deposition numbers CCDC-755860 (**1**) and 755861 (**2**).

**Table 2:** Crystallographic data for **1 **and** 2**.

	1	2
**Empirical Formula**	C_31.25_H_31.25_Cl_2_Mo_2_N_6.25_O_4_	C_27.5_H_29_Cl_3.5_Mo_2_N_5.5_O_4_
**Formula Weight**	821.2	816.5
**Crystal System**	monoclinic	monoclinic
**Space Group**	*P* 2_1_/*c*	*P* 2_1_
***T* (K)**	153(2)	150(2)
***a* (Å)**	17.2529(15)	8.7374(1)
***b* (Å)**	22.279(2)	22.4194(3)
***c* (Å)**	19.5330(18)	16.9125(2)
***α* (deg)**	90	90
***β* (deg)**	113.521(2)	90.278(1)
***γ* (deg)**	90	90
***V* (Å^3^)**	6884.1(11)	3312.90(7)
***D*_calcd_ (g/cm^3^)**	1.585	1.637
***Z***	8	4
***λ* (Å)**	0.71073	0.71073
***μ* (mm^–1^)**	0.928	1.080
**Collected Reflections**	45758	25375
**Unique Reflections, *R*_int_**	16549, 0.1035	14434, 0.0243
**Observed Reflections**	7525	13356
***R*1^[a]^ (*I*>2** ***σ*(*I*))**	0.0525	0.0286
***wR*2^[b]^ (all data)**	0.1230	0.0727

^[a]^*R*1 = ∑||*F*_o_|–|*F*_c_||/∑|*F*_o_|. ^[b]^*wR*2 = {∑[*w*(*F*_o_^2^–*F*_c_^2^)^2^]/∑[w(*F*_o_^2^)^2^]}^1/2^.

## 4. Conclusions 

A neutral [Mo_2_O_4_Cl_2_(Py)_4_] complex represents yet another {Mo_2_O_4_}^2+^ species, obtained from a seemingly simple reaction system of a mononuclear molybdenum(V) starting material (PyH)_5_[MoOCl_4_(H_2_O)]_3_Cl_2_ and pyridine. The updated series includes apart from the latest addition also tetranuclear [Mo_4_O_8_(OMe)_2_Cl_2_(Py)_4_], pentanuclear [Mo_5_O_11_(OMe)_4_(Py)_4_], hexanuclear [Mo_6_O_12_(OMe)_6_(Py)_4_], octanuclear [Mo_8_O_16_(OMe)_8_(Py)_4_], decanuclear [Mo_10_O_26_(Py)_8_] and dodecanuclear [Mo_12_O_28_(OMe)_2_Cl_2_(3-MePy)_8_] clusters. 
